# Ten Simple Rules for Online Learning

**DOI:** 10.1371/journal.pcbi.1002631

**Published:** 2012-09-13

**Authors:** David B. Searls

**Affiliations:** Independent Consultant, Philadelphia, Pennsylvania, United States of America; Whitehead Institute, United States of America

The success of online courseware such as that offered by the Massachusetts Institute of Technology (MIT) (http://ocw.mit.edu) and now by many other institutions, together with a plethora of recent announcements of major new initiatives in this arena such as Coursera (https://www.coursera.org), Udacity (http://www.udacity.com), and the Harvard-MIT partnership edX (http://www.edxonline.org), have made it clear that online learning has reached a tipping point. Many signs point to the possibility in the near future of getting a quality, university-level education at a distance, and for free. As exciting as this prospect may be, it behooves online students to follow a few simple rules for getting the most out of the experience, while being realistic in their expectations, as outlined below.

## Rule 1: Make a Plan

There are many possible motivations for becoming involved in online learning, whether in bioinformatics or any other field. There's nothing wrong with taking an online course on impulse, or to fill a very specific need, or simply for fun, if that's your goal. But if you hope to acquire a broader swath of knowledge for some larger purpose, you will need a directed, organized approach to be efficient and effective, especially in the absence of a formal degree program or traditional academic advisor. Don't underestimate the importance, or the difficulty, of this planning effort, particularly if you are new to the field.

Start by deciding on a curriculum that suits your needs, and determining the optimum sequence of courses. The companion article “An Online Bioinformatics Curriculum” by this author offers a selection of courses and some advice on possible tracks within that particular interdisciplinary field [1]. More generally, professional societies will often publish curriculum recommendations, and yet another approach is to examine university course catalogs (often available online) for their recommended curricula in your area of interest. Do this for several programs that you know to be of high quality, to arrive at a consensus. The particular courses needn't be online—you just need the titles or descriptions so that you can seek out an analogous course that is available to you. Pay attention to the syllabus and listed prerequisites to make sure the online course is at the appropriate level, and that you have adequate preparation. If no online version is available, the same search will allow you to determine an appropriate textbook and/or reading list to make up the gap.

Finally, keep in mind what you hope to achieve at the end. This may be simply knowledge for its own sake, but if you need a set of skills to accomplish some life goal (and in particular if you need it to help you get a job, gain advancement, or qualify for some academic program), that fact should shape not only which courses you take, but how you take them. This will be discussed further in Rule 9.

## Rule 2: Be Selective

We generally try to get best value-for-money in our education, as in all else. As the number of courses available continues to grow, you may have the opportunity to shop around. Be selective in choosing online courses, because in fact there's no such thing as a free course, to the extent that you value your time. Seek out the best institutions, and then seek out the best teachers, just as you would on campus. If you don't already know a professor by reputation, it's easy to do the usual assessment by means of bibliographic search. A web search of a particular course identifier at a major university will typically yield course/professor evaluations by students in various fora, though in practice these have a tendency to generate more heat than light.

The evaluation of a course must also take format into account. In many cases only bare, unannotated videos of lectures are available, as for the UC Berkeley Webcast resource (http://webcast.berkeley.edu), and these can be highly variable in quality and especially in legibility of projected slides or writing on a blackboard or whiteboard. A quick sampling will help you decide if the video quality suits you. If not, try searching for a corresponding course website to see if the slides or detailed course notes are separately available, so that you can have them up while you run the video (or even audio, which in rare instances is the only form of recording available).

In other cases, no video is provided but only detailed syllabi, lecture notes, readings, quizzes, exams, and/or demonstrations (as for the majority of courses on the MIT OpenCourseWare (OCW) project (http://ocw.mit.edu), and innumerable course web sites). To be sure, these materials can be valuable, and in particular there is a growing trend to posting notes in highly polished form and even as full-fledged online textbooks. But video lectures have many advantages: a sense of immediacy, the feeling of a personal touch, helpful emphasis and nuance in the presentation, and the simple fact that a memorable professor makes for memorable subject matter. In such skilled hands, the video format affords the use of techniques that have been shown to enhance learning, including not only visual material but also expressions of enthusiasm by the lecturer and even humor [2].

Having both lecture videos and ancillary course material, as for example with the Open Yale courses to varying degrees (http://oyc.yale.edu), is a huge plus. Even better is the recent trend to structured courseware in which video presentations are modularized and interspersed with assessments and homework. Some of these are even run on a set schedule, so that videos are released and homeworks are due on a regular weekly basis. All else being equal, the courses offered under this model by initiatives such as Coursera and edX should always be preferred.

## Rule 3: Organize Your Learning Environment

Create an environment that will promote learning. In terms of time, a regular schedule (such as those imposed by the structured initiatives) can help ensure that you keep momentum, so that successive learnings reinforce each other before they have a chance to fade. Let your mind get used to a regular workout. And resist the temptation to rush through an online course; cognitive psychologists studying learning have long noted a “spacing effect” that suggests it is better to absorb material at regular, separated intervals than all at once, which is why “cramming” for exams is so ineffective [3].

In terms of your work space, use common sense and give yourself some quiet and privacy to concentrate on lectures. Set up your screen with an enlarged video display together with associated materials such as slides, and close other windows, especially e-mail and the like. Cognitive psychologists now have experimental evidence of the insidious effect of multitasking on the learning process [4]. While online courses often provide detailed lecture notes, it is a good idea to take your own notes anyway, as research has long suggested the benefits of this activity to long-term learning, particularly at deeper levels of understanding [5].

## Rule 4: Do the Readings

There is a whole set of truisms about classroom education that carries over to online education with very little change, except perhaps the need for a greater emphasis when one is on one's own. Particularly important is to prepare for each class as indicated in the syllabus, which generally means completing the required readings ahead of time. It's too easy to neglect this when online, knowing you will never be called on in class by the instructor.

Don't adopt the attitude that the lecture should be a painless way to be spoon-fed the material and that otherwise you may as well just read the textbook on your own. In almost every case the lecturer is doing you the favor of assigning carefully selected readings rather than the entire book, helping you to focus on the “meat” that is most relevant to the approach being taken in the course. Your end of the bargain is to come prepared so that you can most efficiently absorb the value-added of the multimedia presentation.

Many courses include journal articles in their assigned reading, as a teaching tool. Particularly as you reach more advanced stages, it's a good idea to read the current literature on your own as well, not only for the learning experience but because it will reveal to you any gaps in your knowledge or skills. This in turn may prompt you to adjust your selection of courses or independent study. At the end of your efforts, the ability to read and understand the key journals in your field will establish both your competency and currency in the subject.

## Rule 5: Do the Exercises

Another platitude that will come as no surprise is that you should do your assigned homework before you get to watch any TV. You know in your heart that you learn by doing, and once again it's too easy to neglect this when nobody is collecting and grading your work.

For courses that are computational in nature, there is the additional imperative to do the programming assignments faithfully. You haven't really taken a programming course if you haven't been through the hard slogging: designing, testing, debugging, documenting, refactoring, etc. If the assignment is just too dull, you have the luxury of being able to tweak it towards some variation that interests you, but come what may, it's well known that you must put in the time to become a proficient coder.

In fact, if you are doing general programming classes in an interdisciplinary program like bioinformatics, you may want to put in a special effort to modify the programming assignments and projects so that they apply to your domain of interest. You will need experience obtaining and working with the relevant data as much as you need practice with the programming languages and algorithms themselves. It's hard enough being interdisciplinary without doing twice the work, so kill two birds with one stone whenever you can.

## Rule 6: Do the Assessments

Many courses will have quizzes and exams in addition to homework, and once again it is important not to neglect these, for the obvious reason that you need to know how well you are absorbing the material. This is the opportunity to make mid-course corrections, which are far easier to execute in online learning. Moreover, a well-constructed exam can be a learning experience in itself, particularly if it is the occasion for you to whip up your competitive instincts just a bit, since this is likely to improve your retention. Many lines of research point to the benefits of testing in promoting effective learning [6].

The newer structured learning approaches such as Coursera not only institutionalize exams, running them in set intervals, but also integrate quizzes directly into lectures. This format promises to provide feedback not only to the users but to the providers as well, indicating what points need clarification in the associated discussion fora or supplemental short videos. No doubt there will be other ways in which data analytics can be applied to improving the educational experience and effectiveness of these new platforms.

## Rule 7: Exploit the Advantages

There is at least some experimental evidence that online lectures with integrated assessment can produce results superior to the classroom for large courses, possibly by avoiding known issues of “poor attendance… and inappropriate behavior (talking, sleeping, reading) on the part of students who do attend class” [7]. A meta-analysis of ten such comparative studies found that online versions of courses had learning outcomes better than traditional versions in four cases, worse in two, and indistinguishable in four [8]; the authors of this study noted pros and cons to online learning: “The significant pro is the element of convenience which eliminates the constrictive boundaries of space and time. The most notable con involves the impersonal nature of the online environment.”

The disadvantages of online learning will be addressed below. As for the advantages of convenience, first among these is the flexibility you have to schedule classes, in whatever order is appropriate. Most courses today are available to download and view as you see fit, and at a pace that suits your schedule. Gone are the days of missing out on an upper-level elective because it is only offered in alternate years, or the professor is on sabbatical. If you start a course and find you are ill-prepared, you can “drop” the course without prejudice and look for the appropriate prerequisite. While it is true that the newer structured courses are often offered on a set schedule, indications are that the materials will still be available afterwards for individual viewing, though without some of the “live” features.

Even the viewing experience itself offers new possibilities. One can freeze a lecture to work out a knotty point, and back up to repeat sections. Entire lectures can be repeated as needed, for example after any sort of forced hiatus. Many courses recorded in classrooms spend a major portion of the first lecture and parts of others dealing with administrivia, which is easily skipped over. Many video players used in these sites allow for a lecture to be sped up by 1.25× or 1.5×, for lecturers who speak glacially or for segments that are easily grasped. In short, learning online gives the student unprecedented control over the proceedings.

## Rule 8: Reach Out

It is widely believed that the greatest disadvantage of online learning relates to its isolated and impersonal nature [9]. This perspective is ironic given that the Internet is also known as the ultimate medium for social networking. In fact, the newer structured approaches like Coursera do have discussion forums that allow students to commune with one other, which in addition are monitored by instructors who can post authoritative responses to questions and even alter elements of the course as needed. Take advantage of these features instead of just lurking. If you are taking a course without such a built-in community, it will take more work, but if you can locate or recruit other like-minded students you may find that you learn better in a group, however small.

A more difficult challenge is the absence of a live instructor or tutor with whom you can directly interact. Having someone to coach you through a difficult concept, targeting your specific needs, is an advantage of traditional education that is obviously lacking in recorded lectures. Research is underway in adaptive instructional systems [10] that may eventually contribute to the new online learning initiatives, but in the meantime you must make the best of it.

For some, the most valuable feature of live instruction may not be the continual give-and-take so much as the ability to get past some particular roadblock to understanding. In such cases one can always try contacting the course instructor with a very specific question, though obviously you should not be surprised if this tactic fails. You can also try cold-mailing other teachers, researchers, or experts, preferably with some connection to you. In the end, though, you are most likely going to puzzle it out for yourself, through extra reading and online search. Don't be too discouraged by this, because the time and effort you put in will make it more likely that you retain the concept and understand it at a deeper level; educational psychologists call this “productive failure” [11].

## Rule 9: Document Your Achievements

A somewhat cynical view of traditional higher learning is that its value lies in the diploma as much as the knowledge. Certainly the lack of a diploma from any self-study program, online or otherwise, can be an impediment to getting the opportunity to apply one's hard-won knowledge. To a potential employer or a graduate admissions office, for example, a diploma and a transcript are proof of sorts that you know what you say you know.

In its current state, open online learning simply doesn't have an equivalent mechanism. Recent programs have taken to offering certificates of completion, but these are carefully distinguished from actual course credits at the sponsoring institution. There are several movements to establish formal systems of so-called badges indicating skills and achievements acquired from activities like online learning, particularly in computational circles, for instance in the case of Mozilla Open Badges (http://openbadges.org). Any of these may or may not carry some weight in the outside world, depending on a given employer's or university's trust in both the effectiveness and the integrity of the online resource, but they certainly don't approach the luster of a degree from an accredited school.

Whether or not any sort of certification is connected with courses you take, if you want to demonstrate that you have learned the material you should take concrete steps from the outset to document this in any way you can. Record your program of study in something resembling a transcript, including brief course descriptions, institutions, and dates of completion. Create a portfolio of accomplishments, including reading lists, major exams, and any substantial homework assignments; while it may not be possible to prove definitively that you came by them honestly, it will suggest that you were serious about your studies. Perhaps add some work on your own initiative, such as essays or projects based on learnings from the course. For computational courses in particular, assemble your programming assignments and projects, making sure that the code is impeccably documented and presentable. Even if such a portfolio of accomplishments lacks the imprimatur of a formal diploma, it is a good habit of mind and can be motivational as well.

## Rule 10: Be Realistic

It is important to be realistic in your expectations of online learning. As Rules 3–6 suggest, the single most important factor in your success at learning will be your degree of motivation, which in turn will determine your receptiveness and work ethic. While motivation is also necessary for success in a traditional campus environment, there is no doubt that learning on your own requires a special brand of persistence, and is not for everyone. Don't set yourself up for disappointment by mistaking free courseware for a free lunch. On the other hand, a casual attitude is fine as long as it aligns with your expectations.

Be cognizant, also, of the inherent limits of online learning. As discussed in Rule 8, without live instructors and tutors you will encounter various kinds of hurdles that, however, may be overcome with extra effort. What is harder to surmount is the lack of an academic advisor to guide you on the broader issues. Rule 1 offers general advice on curriculum planning, but true mentorship, extending to career advice, is harder to come by. Jump at every chance you get to ask experts for guidance, however fragmentary, and pay attention to the literature to be sure you are studying what is really called for.

Other limits to online education include the lack of certain kinds of training, such as laboratory experience and presentation skills. These shortcomings may be a bit less critical in some areas, such as computer science, but they will loom larger in fields like molecular biology. In such cases you will need to use your imagination to fill the gap; for instance, you might seek unpaid internships or consider paying for some carefully selected traditional classes.

In the final analysis the problem of certification raised in Rule 9 may be the single biggest practical issue facing the online learner. John F. Kennedy, upon being awarded an honorary degree from Yale, said that “now… I have the best of both worlds, a Harvard education and a Yale degree” [12]. One is left to wonder how he would have fared with a Harvard education and neither degree. If you are happy to have the knowledge without the diploma, then you may rest easy on this point, but otherwise you need to do a careful cost-benefit analysis before investing time in an online education.

While it is important to be realistic about all these factors, don't let it dampen your enthusiasm. With the steadily increasing momentum behind online learning, it is more than likely that the initiatives themselves will begin to address the issue of effective certification, and perhaps even that of mentorship, even as the catalog of available courses expands rapidly. Anyone beginning a course of online study today is likely to find a greatly changed landscape even by the time they complete their education.
